# Meat Nutritional Value of Puławska Fattening Pigs, Polish Large White × Puławska Crossbreeds and Hybrids of DanBred

**DOI:** 10.3390/ani13152408

**Published:** 2023-07-26

**Authors:** Grzegorz Siemiński, Piotr Skałecki, Mariusz Florek, Piotr Domaradzki, Ewa Poleszak, Małgorzata Dmoch, Małgorzata Ryszkowska-Siwko, Monika Kędzierska-Matysek, Anna Teter, Marek Kowalczyk, Agnieszka Kaliniak-Dziura

**Affiliations:** 1 Department of Quality Assessment and Processing of Animal Products, Faculty of Animal Sciences and Bioeconomy, University of Life Sciences in Lublin, Akademicka 13, 20-950 Lublin, Poland; grzegorz.sieminski@up.lublin.pl (G.S.); piotr.domaradzki@up.lublin.pl (P.D.); malgorzata.dmoch@up.lublin.pl (M.D.); malgorzata.siwko@up.lublin.pl (M.R.-S.); monika.matysek@up.lublin.pl (M.K.-M.); anna.teter@up.lublin.pl (A.T.); marek.kowalczyk@up.lublin.pl (M.K.); agnieszka.kaliniak@up.lublin.pl (A.K.-D.); 2 Chair and Department of Applied and Social Pharmacy, Medical University of Lublin, Chodźki 1, 20-093 Lublin, Poland; ewa.poleszak@umlub.pl

**Keywords:** pork, breed, nutritional value, proximate composition, amino acids, fatty acids

## Abstract

**Simple Summary:**

The old indigenous breeds of pig kept in Poland are currently an important element in the preservation of livestock biodiversity. In turn, the basis for the commercial production of fattening pigs are inter-breed hybrids. Today, one of the most important challenges facing pig producers is to obtain meat with specific nutritional and taste qualities. The aim of this study was a comparative analysis of the nutritional quality of meat and the health-promoting properties of intramuscular fat from pigs of the native Puławska breed and its crossbreeds with the Polish Large White breed and the DanBred hybrids. Significantly, the meat of pigs of the Puławska breed despite the lowest intramuscular fat content contained the lowest amount of saturated fatty acids, showed the most favourable PUFA/SFA ratio and the best values of atherogenic and thrombogenic indices compared to both other genetic groups. Meat from the fattening pigs of the Puławska breed also tended to have a high protein content with the highest nutritional quality index value and a high concentration of indispensable amino acids, including histidine.

**Abstract:**

The nutritional quality of meat and the health-promoting properties of intramuscular fat from randomly selected rearing pigs of the native Puławska breed (PUL, *n* = 15) and its crossbreeds with the Polish Large White breed (PLW × PUL, *n* = 16) and the DanBred hybrids (DAN, *n* = 17) were compared. The typical commercial fattening period was carried out up to 80 days of age. The initial body weight of PUL weaners was 30.55 kg (±3.98 kg), that of PLW × PUL weaners was 30.00 kg (±4.29 kg) and that of DAN weaners was 30.70 kg (±3.40 kg). The protein content and energy value of the complete feeds (Grower and Finisher) were 165 and 155 g/kg, and 12 and 11.8 MJ/kg, respectively. The research material consisted of samples of *Longissimus lumborum* and *Semimembranosus*. The chemical analyses included the determination of proximate composition, fatty acid content and amino acid concentration. The lowest fat content was found in the meat of the Puławska pigs, while the highest was found in the DanBred fattening pigs. The highest content of SFAs and MUFAs was found in the meat of DanBred hybrids, while it was significantly lower in Puławska pigs. The genetic group did not affect the content of PUFAs, including *n*-3 and *n*-6. Significantly lower and more favourable atherogenic and thrombogenic indices were found for the intramuscular fat of Puławska pigs. The very limited effect of the genetic group on amino acid content was observed, except for that of serine, histidine and lysine. In general, the meat of pigs of the Puławska breed showed the highest nutritional value and the most favourable health-promoting properties.

## 1. Introduction

The average per capita consumption of pork in EU countries in 2021 was 32.5 kg, while in Poland it was 43.4 kg [1]. Unfortunately, it is predicted that pork production will be reduced in the European Union by 2030 due to environmental and animal welfare concerns which will reduce the domestic demand for pork [2]. In turn, it is estimated that the growth in demand for pork and pork products worldwide will increase by more than 37% by 2050 [3]. As consumer preferences are constantly changing [4] on the pork market, a number of measures are being taken to improve the quality of the raw material along genetic lines [5], while taking into account the entire pig production system [6], including welfare optimisation [7]. All these measures are aimed at eliminating the stress factors that contribute significantly to the occurrence of defective meat [8]. 

In Poland, five domestic breeds are maintained in pedigree breeding: the Polish Landrace (PL), the Polish Large White (PLW), the Puławska (PUL), the Złotnicka White (ZW) and the Złotnicka Pstra (ZP) breeds, the latter three being native breeds [9]. PLW pigs are characterised by a high level of reproductive performance traits, a relatively high meat yield and good feed conversion rates [10]. The Puławska breed is the oldest indigenous breed of pig kept in Poland and is currently an important element in the preservation of livestock biodiversity. Such action is consistent with the current needs of consumers, who prefer pork and pork products with high nutritional value and organoleptic qualities [11].

At present, inter-breed hybrids are the basis for the commercial production of rearing pigs. In Poland, commodity hybrids are obtained by crossbreeding sows of domestic maternal breeds (PLW, PL or their hybrids) with the paternal component, which are boars of the Duroc, Pietrain, Hampshire, Belgian Landrace and line 990 breeds [12]. Crossbreeding involving Landrace, Yorkshire and Duroc pigs is also practised worldwide [13].

Selecting the right breeds, breeding lines or specific genotypes for pig livestock production is a fundamental factor in determining the efficiency of pig production [14]. Knowing the broadly understood meat quality of different breeds and lines of pigs and their ability to produce high-quality pork is an important challenge for both pig producers and meat processors [15]. Therefore, innovative solutions are being sought to produce pork with specific nutritional and taste qualities. One such solution is to cross-breed conservation breeds of pigs with contemporary breeds, which makes it possible to obtain carcasses of crossbreeds with a higher dietary value of meat [16]. Therefore, the aim of this study was a comparative analysis of the nutritional quality of meat and the health-promoting properties of intramuscular fat from pigs of the native Puławska breed and its crossbreeds with the Polish Large White breed and the DanBred hybrids. 

## 2. Materials and Methods

### 2.1. Animals

The research was carried out at one of the experimental and production farms of the University of Life Sciences in Lublin in the course of two fattening in one production cycle (2018–2019) with a total of 420 animals. Ethical review and approval were waived for this study (see Institutional Review Board Statement). Piglets were kept in one pen (1 unit/1 m^2^ area) with equal access to feed and water. The conditions for housing the pigs, as well as the requirements for hygienic indicators (air circulation, dust level, temperature, relative humidity and gas concentration) to ensure proper animal welfare were in line with EU [17] and national legislations [18] in this respect and were controlled by the Veterinary Inspectorate. Pigs were fed in two phases with grower and finisher complete feeds. The nutrient content (in g/kg) and energy value of the feeds (MJ/kg) used in the fattening period are shown in Table 1. The study included 48 rearing pigs (hogs) randomly selected for fattening representing three genetic groups, i.e., pigs of the native Puławska breed (pure-bred specimens, *n* = 15, PUL), two cross-hybrids (Polish Large White ♂ × Puławska♀, PLW × PUL; *n* = 16) and DanBred commercial hybrid breeds (DanBred Landrace × DanBred Yorkshire, DAN, *n* = 17). The average body weight of all piglets was 30.4 kg; in the PUL group it was 30.55 kg (±3.98 kg), in the PLW × PUL group it was 30.00 kg (±4.29 kg) and in the DAN group it was 30.70 kg (±3.40 kg). At the end of the typical commercial fattening period (at 80 days of age), the animals were transported to the slaughter plant via the road in accordance with the veterinary requirements of Regulation (EC) 1/2005 [19]. 

The average pre-slaughter weight of the animals in each group was, respectively 84.21 kg ± 3.31 for PUL, 92.44 kg ± 4.03 for PLW × PUL and 106.01 kg ± 6.19 for DAN. After 24 h of rest, the rearing pigs were slaughtered in accordance with the provisions of Council Regulation (EC) No. 1099/2009 of 24 September 2009 on the protection of animals at the time of slaughtering [20]. Carcasses were stored for 24 h under refrigerated conditions, 2 °C (±1 °C). The research material for chemical analyses consisted of samples of 2 skeletal muscles (at a weight of 500–750 g), i.e., those of *Longissimus lumborum* (LL, section L1–L5) and *Semimembranosus* (SM). Vacuum-packed samples were transported in compressor thermochambers maintaining the temperature of the meat at 2 °C (±2 °C).

### 2.2. Chemical Analyses

The basic chemical composition of the muscles was determined via reference methods, i.e., water content was determined via the drying method (103 °C) in accordance with PN-ISO 1442:2000; ash content was determined via the muffle furnace ashing method (550 °C) in accordance with PN-ISO 936:2000; total protein content was determined via the Kjeldahl method using a Büchi B-324 apparatus in accordance with PN-A-04018:1975/Az3:2002; fat content was determined via the Soxhlet method (*n*-hexane) using a Büchi B-811 apparatus in accordance with PN-ISO 1444:2000. The energy value in kJ of a 100 g portion of meat was calculated on the basis of total protein and fat, assuming physical energy equivalents (protein: 23.64 kJ; fat: 39.54 kJ). The Nutritional Quality Index (NQI) for protein and fat was calculated according to Hansen et al. [21], taking into account the reference intake values for energy and nutrients in accordance with Regulation (EU) No 1169/2011 [22].

The fatty acid content after previous fat extraction [23] was determined in accordance with PN-EN ISO 12966-2:2011 and PN-EN ISO 5508:1996. Fatty acid methyl esters were determined via gas chromatography using a Varian GC 3900 (Walnut Creek, CA, USA) with a flame ionisation detector (FID) and a CP 7420 capillary column (100 m long; 0.25 mm internal diameter; 0.25 μm film thickness). The analysis was carried out under variable temperature conditions (an initial column oven temperature of 50 °C, final temperature of 260 °C, and dispenser and detector operating temperature of 270 °C). The flow rate of the carrier gas (hydrogen) was 2 mL/min, that of air was 300 mL/min and that of the make-up was 30 mL/min. The volume of dispensed sample was 1 μL, and the partition coefficient was 1:50. Identification of the fatty acids was carried out on the basis of their relative retention times compared to those of the fatty acid methyl ester standards (Supelco Inc., Bellafonte, PA, USA). The identification of fatty acids was carried out in accordance with EN ISO 5508:1996, using the computer programme Star GC Workstation Version 5.5 (Varian Inc., Walnut Creek, CA, USA). A total of 27 fatty acids were identified; however, in the tables, the 9 most important FAs are presented. The tables show the results as content in mg/100 g of muscle tissue for selected fatty acids and the sum of the individual groups: saturated (SFA), monounsaturated (MUFA) and polyunsaturated fatty acids (PUFA), broken down into FA *n*-3, *n*-6 and *n*-9. In addition, the following ratios and indices were calculated: PUFA/SFA, *n*-6/*n*-3, AI—atherogenic index [sum of C12:0, (4 × C14:0) and C16:0]/[sum of MUFA, *n*-6 and *n*-3], and TI—thrombogenic index (sum of C14:0, C16:0 and C18:0)/[sum of (0.5 × MUFA), (0.5 × *n*-6), (3 × *n*-3) and (*n*-3/*n*-6)] [24].

Amino acid content was determined using Automatic Amino Acid Analyzer AAA 400 (Ingos Ltd., Prague, Czech Republic). Amino acids were separated via ion exchange chromatography. A 0.37 cm × 45 cm column was filled with an ion exchanger in the form of a resin. The column temperatures were 60 °C and 74 °C. The apparatus identified the amino acids via a reaction with ninhydrin (detection reagent). Separation of the amino acids was carried out with a photometric detector at 570 nm (for proline 440 nm). Acid hydrolysis of proteins was performed following the method of Davis and Thomas [25], using 6 N HCl. The hydrolysate was evaporated on a vacuum evaporator and then dissolved in citrate buffer (pH 2.2). The prepared sample, after filtering through a syringe filter, was dispensed onto an amino acid analyser column. Seventeen amino acids were included in the analysis, Asp, Thr, Ser, Glu, Pro, Gly, Ala, Cys, Val, Met, Ile, Leu, Tyr, Phe, His, Lys, and Arg, for which the results are expressed in mg/g of protein.

### 2.3. Statistical Analysis

The data obtained were statistically processed with a one-way analysis of variance using STATISTICA v. 13 (TIBCO Software Inc., Palo Alto, CA, USA). The pre-approved statistical model did not confirm a significant effect of year (2018 vs. 2019) within each group of rearing pigs, so the analysis was limited to a one-factor analysis of variance examining the effect of the genetic group on the nutritional value parameters of the assessed skeletal muscles. The results are given as mean values for the individual traits and the standard error of the mean. The significance of differences for individual scores was calculated using the Duncan test (*p* ≤ 0.05 and *p* ≤ 0.01).

## 3. Results and Discussion

### 3.1. Proximate Composition 

No significant differences were found in the moisture (water) content, ash content and energy value of the meat of the genetic groups of the evaluated pigs. However, significant differences were found in intramuscular fat content (Table 2). The lowest (*p* < 0.01) fat content was found in both muscles of the Puławska breed rearing pigs (1.36% in LL and 1.50% in SM), while the highest was found in the DanBred fattening pigs (2.67% in LL and 2.91% in SM). It is worth mentioning that there was a trend (*p* < 0.1) of higher protein content in meat from the pigs of Puławska breed compared to that of the meat from the rearing pigs of the other groups. Consequently, the meat from the Puławska breed was characterised by the (*p* ≤ 0.01) highest value of the NQI for protein and with the (*p* ≤ 0.01) lowest value for fat. The average moisture content of both evaluated muscles, regardless of genetic group, was within the range typical of meat from domestic breeds of pigs (73.1–75.3%) [9]. Babicz et al. [26] found in the meat of fattening pigs of the Puławska breed a chemical composition similar to that obtained in the presented study for both m.l.l. and m.sm. Kasprzyk and Bogucka [27] reported a higher fat content (2.73%) but lower protein content (22.90%) in *Longissimus lumborum* of fattening pigs of the Puławska breed, while for DanBred hybrids they obtained a similar level of fat (2.50%) to that in their study and a lower protein content (20.85%) compared to that in their study, although it should be noted that the quoted results were obtained for pigs with slaughter weights ranging from 103 to 105 kg. For PUL × PLW hybrids fattened for 99 days to a weight of 103.4 kg, Milczarek et al. [14] report a fat content in the loin (*Longissimus dorsi*) of 1.76% and a protein content of 23.70%, while in ham (*Adductor*) it was 2.11% and 22.81%, respectively, i.e., there was less fat and more protein compared to the values obtained in their own study for this group of hybrids of native breeds.

In Poland, it is assumed that the intramuscular fat content required for the optimum taste and flavour of meat should be between 2.5 and 3% [28,29]. In the study presented here, particularly in the meat of pigs of the Puławska breed, the level of intramuscular fat was significantly lower than recommended, which was related to the slaughter weight being too low for this native breed. However, previous studies have shown that meat from the pigs of the PUL breed with typical intramuscular fat content showed good oxidative stability and was suitable for 14 days of maturing under vacuum conditions [30]. 

### 3.2. Fatty Acids 

The nutritional value of fat is determined primarily by the type and content of the individual fatty acid groups. In LL, the genetic group significantly differentiated the content of major saturated fatty acids (C14:0, C16:0 and C18:0) and their total content, as well as the content of monounsaturated FAs (C16:1*n*-7 and C18:1*n*-9) and their sum (Table 3). Identical relationships were also found in the SM (Table 4). Indeed, the highest content of SFAs and MUFAs was found in the LL of DAN hybrids, while it was 2–2.5 times lower in PUL pigs, which is particularly favourable for proatherogenic FAs such as myristic and palmitic FAs. In contrast, the genetic group did not significantly affect the content of polyunsaturated FAs, including the *n*-3 and *n*-6 PUFAs, in both of the muscles evaluated. The fatty acid content of LL was not significantly different between PUL pigs and PLW × PUL hybrids (Table 3), while in SM all differences (except C20:1*n*-9) were found to be significant. 

Milczarek et al. [14] showed no significant differences in the fatty acid profile of the loin and ham of domestic hybrid breeds (Puławska × Polish Large White and Polish Landrace × Polish Large White). However, many authors indicate that the breed of pig significantly differentiates the fatty acid composition of muscle tissue [31], with meat from slower-growing pigs showing a more favourable fatty acid profile, manifesting in particular as a higher proportion of PUFAs [32].

Currently, a number of indicators are used to assess the health-promoting values of fats and the potential beneficial effects of their consumption in humans [33]. The lower SFA content in intramuscular fat of LL (Table 3) and SM (Table 4) of PUL pigs determined a significantly (*p* ≤ 0.05) more favourable PUFA/SFA ratio (0.20 and 0.30, respectively) compared to that of PLW × PUL hybrids (0.14 and 0.17) and DAN hybrids (0.06 and 0.18), although it did not reach the recommended level of 0.4 [34]. In contrast, Milczarek and Osek [35] in a study on PUL pigs fattened to a weight of 116 kg obtained a much lower value for the PUFA/SFA ratio, i.e., that for LL was in the range of 0.057–0.064, and that for SM was 0.055–0.058. Higher values for this ratio (in the range of 0.36–0.50) were reported by Choi et al. [13] for the *Longissimus thoracis* (LT) of purebred pigs and LYD (Landrace × Yorkshire × Duroc) three-breed hybrids with a slaughter weight of 110–114 kg. 

In the present study, the PUFA *n*-6/*n*-3 intramuscular fat ratios of all genetic groups assessed were found to be very high. Choi et al. [13] report values of this ratio similar to those obtained in the present study for the *Longissimus thoracis* of purebred pigs (about 20.7) and LYD three-breed hybrids (about 18.6). Significantly lower levels of the *n*-6/*n*-3 ratio are reported by Kasprzyk et al. [36] for the *Longissimus thoracis* and *lumborum* (LTL) of PUL breed fattening pigs (12.68) and PLW pigs (15.61). It is worth noting that, despite the widespread use of this indicator to assess the health-promoting value of lipids, it is currently subject to criticism pointing to its theoretical and practical limitations [37,38]. 

Significantly (*p* ≤ 0.05) lower and thus more favourable values of the atherogenic and thrombogenic indices were found for the intramuscular fat of both PUL pig muscles evaluated (0.50 and 1.24 for LL and 0.46 and 1.13 for SM, respectively) compared to that of the other genetic groups, which was related to the several-fold lower content of saturated acids, especially C14:0 and C16:0 (Table 3 and Table 4). For the *Longissimus dorsi* of DanBred × PIC terminal line hybrids, Alvarenga et al. [39] reported PUFA/SFA ratio values ranging from 0.46 to 0.48, *n*-6/*n*-3 ratio values ranging from 2.25 to 2.80 and AI values ranging from 0.27 to 0.31. Kasprzyk et al. [36], for lipids from the LTL of PUL fattening pigs, obtained an average AI value of 0.46 and TI value of 1.12, while for PLW pigs they obtained values of 0.46 and 1.14, respectively. Although different AI and TI levels for fats with potential beneficial effects on human health are reported in the literature, no values for these indices are currently officially recommended [33]. It is generally accepted that consumption of lipid-containing foods characterised by lower levels of AI and TI can reduce the risk of coronary heart disease [24]. 

### 3.3. Amino Acids

Statistical analysis of the amino acid content of the protein from the skeletal muscles assessed showed a very limited effect of the genetic group of the rearing pigs. In the case of LL (Table 5), significant (*p* ≤ 0.05) differences were found in serine, histidine and lysine content, with the highest lysine and serine content being found in PLW × PUL hybrids and the highest histidine content being found in PUL pigs. The protein of the fattening pigs of these two breed groups also contained the most IAA (indispensable amino acids).

In contrast, for the semimembranosus muscle, significantly (*p* ≤ 0.01), the most histidine was found in PUL pigs (Table 6), which also had the highest IAA content. 

In general, the amino acid composition of meat from domestic animals has been known for a long time [40], obviously subject to modifications related to the influence of factors such as species, breed, age or muscle location, and processing [41]. In their analysis of amino acid content of the longissimus muscle of pigs of two genetic lines (faster-growing G1, with low fat, vs. slower-growing G2, with a higher fat content), Wilkinson et al. [42] found significant differences only for serine, isoleucine and histidine. In contrast, Zhang et al. [43] showed a significant variation in the amino acid content of the longissimus muscle of five pig breeds most commonly consumed in China. Comparing the results of the amino acid composition obtained in the presented study for LL with those reported by the cited authors, it can be concluded that the Chinese pork was characterised by a lower content of Glu (32.5–37.4), Ala (11.6–13.2), Pro (6.7–7.9), Thr (9.5–11.0), Val (10.1–11.5), Met (5.9–6.8), Tyr (8.3–9.4) and Phe (8.8–10.0). The range of the other amino acids (except for the higher concentration of His 9.8–12.2) was very similar to that in the results obtained in the presented study. 

## 4. Conclusions

A comparative analysis of selected indices of nutritional quality of two skeletal muscles from fattening pigs of the native Puławska breed and their hybrids with the Polish Large White breed and the DanBred hybrid breed showed a significant effect of genetic group on the content of lipid fraction and its health-promoting properties. Despite the lowest proportion of intramuscular fat in the meat of pigs of the Puławska breed, it was significantly characterised by the lowest content of saturated fatty acids, the most favourable PUFA/SFA ratio and the best values of atherogenic and thrombogenic indices compared to those of both other genetic groups. Meat from the fattening pigs of the Puławska breed also tended to have a high protein content with the highest nutritional quality index value (NQI > 6) and a high concentration of IAA, including histidine.

## Figures and Tables

**Table 1:** Nutrient content (g/kg) and energy value (MJ/kg) of the feeds used on the farm.

Ingredient	Grower	Finisher
Protein	165.0	155.0
Fat	34.0	29.0
Crude fibre	49.0	51.0
Crude ash	43.0	44.0
Lysine	10.2	10.1
Methionine	3.3	3.0
Assimilable phosphorus	3.2	3.2
Calcium	6.3	6.3
Sodium	1.5	1.9
Energy	12.0	11.8

**Table 2:** Chemical composition (%) and energy values (kJ/100 g) of *Longissimus lumborum* and *Semimembranosus* of rearing pigs according to genetic group.

Specification	PUL	PLW × PUL	DAN	SEM	*p*-Value
*Longissimus lumborum*					
Moisture	73.77	75.17	74.45	0.28	0.5761
Protein	24.02	22.90	22.34	0.37	0.0890
Fat	1.36 ^A^	2.09 ^B^	2.67 ^C^	0.22	0.0001
Ash	1.17	1.17	1.16	0.12	0.1250
Energy	417.23	447.42	461.28	8.33	0.0570
NQI Protein	6.56 ^C^	6.18 ^B^	5.94 ^A^	0.04	0.0001
NQI Fat	0.24 ^A^	0.40 ^B^	0.50 ^C^	0.02	0.0001
*Semimembranosus*					
Moisture	74.26	75.42	74.86	0.41	0.2599
Protein	23.86	21.52	20.83	0.56	0.0912
Fat	1.50 ^A^	2.80 ^B^	2.91 ^B^	0.23	0.0001
Ash	1.24	1.18	1.17	0.01	0.1223
Energy	420.57	461.84	459.13	9.38	0.1264
NQI Protein	6.41 ^B^	5.88 ^A^	5.81 ^A^	0.10	0.0001
NQI Fat	0.30 ^A^	0.53 ^B^	0.56 ^B^	0.04	0.0001

^A,B,C^ Mean values denoted by different letters in the rows are statistically significantly different at a given *p*-value. PUL, Puławska breed; PLW × PUL, Polish Large White × Puławska; DAN, DanBred hybrids. NQI—nutritional quality index.

**Table 3:** Content of selected fatty acids (mg/100 g of muscle tissue) of intramuscular fat of the *Longissimus lumborum* of the rearing pigs in relation to genetic group.

Specification	PUL	PLW × PUL	DAN	SEM	*p*-Value
C14:0	14.40 ^A^	28.15 ^AB^	35.70 ^B^	26.08	0.0043
C16:0	280.80 ^A^	460.75 ^AB^	686.75 ^B^	78.25	0.0064
C18:0	136.80 ^a^	213.80 ^a^	376.65 ^b^	45.77	0.0123
∑SFA	452.50 ^A^	726.45 ^A^	1118.80 ^B^	128.03	0.0077
C16:1*n*-7	41.65 ^a^	74.90 ^b^	79.95 ^b^	8.86	0.0110
C18:1*n*-9	535.80 ^A^	787.00 ^AB^	1179.05 ^B^	126.51	0.0080
C20:1*n*-9	2.55	3.20	5.70	0.59	0.0753
∑MUFA	584.80 ^A^	871.85 ^AB^	1265.75 ^B^	134.75	0.0080
C18:2*n*-6	84.05	92.80	111.90	7.36	0.1273
C18:3*n*-3	3.80	4.55	5.10	0.33	0.1784
C20:2*n*-6	2.15	2.00	3.20	0.32	0.9800
∑PUFA	92.00	101.15	59.20	14.36	0.1333
PUFA/SFA	0.20 ^b^	0.14 ^ab^	0.06 ^a^	0.03	0.0320
*n*-3	4.50	5.25	5.70	5.15	0.3722
*n*-6	87.45	96.40	116.95	7.55	0.1325
*n*-9	540.45 ^A^	791.40 ^AB^	1183.05 ^B^	126.74	0.0080
*n*-6/*n*-3	19.82	18.33	20.28	4.90	0.6351
AI	0.50 ^a^	0.59 ^b^	0.63 ^b^	0.02	0.0150
TI	1.24 ^a^	1.40 ^b^	1.62 ^c^	0.07	0.0147

^a,b,c,A,B^ Mean values denoted by different letters in the rows are statistically significantly different at a given *p*-value. AI—atherogenic index [(sum of C12:0, 4 × C14:0 and C16:0)/(sum of MUFA cis, *n*-6 and *n*-3)]. TI—thrombogenic index [(sum of C14:0, C16:0 and C18:0)/(sum of 0.5 × MUFA cis, 0.5 × *n*-6, 3 × *n*-3 and *n*-3/*n*-6)]. PUL, Puławska breed; PLW × PUL, Polish Large White × Puławska; DAN, DanBred hybrids.

**Table 4:** Content of selected fatty acids (mg/100 g of muscle tissue) of intramuscular fat of the *Semimembranosus* of the rearing pigs in relation to genetic group.

Specification	PUL	PLW × PUL	DAN	SEM	*p*-Value
C14:0	11.50 ^a^	39.00 ^b^	38.00 ^b^	5.77	0.0373
C16:0	225.00 ^a^	662.00 ^b^	714.00 ^b^	99.80	0.0311
C18:0	107.00 ^A^	301.00 ^B^	370.50 ^B^	51.28	0.0097
∑SFA	370.00 ^a^	1039.00 ^b^	1157.50 ^b^	158.15	0.0260
C16:1*n*-7	34.50	111.00	88.50	14.72	0.1362
C18:1*n*-9	444.00 ^a^	1229.50 ^b^	1326.50 ^b^	180.34	0.0439
C20:1*n*-9	3.50 ^A^	6.50 ^AB^	8.50 ^B^	1.01	0.0123
∑MUFA	485.50 ^a^	1358.50 ^b^	1432.00 ^b^	196.22	0.0330
C18:2*n*-6	104.50	161.50	194.50	19.24	0.3545
C18:3*n*-3	4.50	8.50	10.00	1.26	0.3443
C20:2*n*-6	2.50	3.50	3.50	0.31	0.2810
∑PUFA	114.00	177.50	211.00	20.93	0.5672
PUFA/SFA	0.30 ^B^	0.17 ^A^	0.18 ^A^	0.03	0.0038
*n*-3	4.15	4.50	10.50	1.88	0.2633
*n*-6	109.00	167.50	201.00	19.76	0.5610
*n*-9	448.00 ^a^	1238.00 ^b^	1337.50 ^b^	181.64	0.0465
*n*-6/*n*-3	21.33	18.76	19.11	1.07	0.4450
AI	0.46 ^A^	0.53 ^B^	0.53 ^B^	0.02	0.0025
TI	1.13 ^a^	1.29 ^b^	1.32 ^b^	0.04	0.0367

^a,b,A,B^ Mean values denoted by different letters in the rows are statistically significantly different at a given *p*-value. AI—atherogenic index [(sum of C12:0, 4 × C14:0 and C16:0)/(sum of MUFA cis, *n*-6 and *n*-3)]. TI—thrombogenic index [(sum of C14:0, C16:0 and C18:0)/(sum of 0.5 × MUFA cis, 0.5 × *n*-6, 3 × *n*-3 and *n*-3/*n*-6)]. PUL, Puławska breed; PLW × PUL, Polish Large White × Puławska; DAN, DanBred hybrids.

**Table 5:** Content of amino acids (mg/g) in the *Longissimus lumborum* of rearing pigs according to genetic group.

Specification	PUL	PLW × PUL	DAN	SEM	*p*-Value
Asp	22.90	23.90	22.50	0.31	0.1582
Thr	12.00	12.30	11.75	0.14	0.3550
Ser	9.66 ^ab^	9.82 ^b^	9.31 ^a^	0.11	0.0362
Glu	38.80	40.55	38.00	0.55	0.1248
Pro	9.91	9.91	9.87	0.12	0.9930
Gly	9.99	10.15	9.99	0.05	0.3089
Ala	14.05	14.40	13.75	0.18	0.4347
Cys	3.86	3.99	3.63	0.08	0.4892
Val	12.20	12.40	11.75	0.17	0.3169
Met	8.26	8.93	7.99	0.19	0.2389
Ile	11.15	11.10	10.90	0.11	0.7335
Leu	20.05	20.75	19.55	0.26	0.1509
Tyr	9.71	9.58	9.32	0.11	0.4498
Phe	10.15	10.45	10.05	0.14	0.5924
His	10.90 ^b^	9.04 ^a^	10.30 ^ab^	0.37	0.0315
Lys	20.20 ^ab^	20.95 ^b^	19.85 ^a^	0.22	0.0318
Arg	15.30	15.85	15.05	0.21	0.3374
Total IAA	115.89	116.75	112.62	1.18	0.4028
% IAA	49.00 ^B^	48.38 ^A^	48.74 ^AB^	0.12	0.0010

^a,b,A,B^ Mean values denoted by different letters in the rows are statistically significantly different at a given *p*-value. IAA—indispensable amino acids. PUL, Puławska breed; PLW × PUL, Polish Large White × Puławska; DAN, DanBred hybrids.

**Table 6:** Content of amino acids (mg/g) in the *Semimembranosus* of rearing pigs according to genetic group.

Specification	PUL	PLW × PUL	DAN	SEM	*p*-Value
Asp	22.60	20.95	20.85	0.43	0.1616
Thr	11.65	10.80	10.70	0.24	0.2064
Ser	9.55	8.71	8.82	0.19	0.1391
Glu	37.95	34.90	34.65	0.83	0.2093
Pro	9.40	8.86	9.28	0.13	0.2336
Gly	9.98	9.31	9.47	0.15	0.1437
Ala	13.80	12.80	12.90	0.25	0.1903
Cys	3.75	3.63	3.43	0.07	0.4742
Val	11.75	11.15	11.00	0.21	0.3699
Met	8.11	7.65	7.17	0.17	0.1991
Ile	10.60	9.34	9.79	0.24	0.4274
Leu	19.70	18.30	18.30	0.38	0.2501
Tyr	9.59	8.81	8.86	0.19	0.1778
Phe	9.85	9.48	9.45	0.14	0.5638
His	11.10 ^B^	9.41 ^A^	9.55 ^A^	0.37	0.0315
Lys	19.95	18.70	18.70	0.33	0.2076
Arg	15.15	14.02	14.00	0.29	0.1619
Total IAA	113.53	105.44	104.68	2.12	0.1551
% IAA	48.95 ^b^	49.05 ^b^	48.77 ^a^	0.06	0.0419

^a,b,A,B^ Mean values denoted by different letters in the rows are statistically significantly different at a given *p*-value. IAA—indispensable amino acids. PUL, Puławska breed; PLW × PUL, Polish Large White × Puławska; DAN, DanBred hybrids.

## Data Availability

All data are available on request from authors.
